# *Haemophilus influenzae* Pyomyositis in a Patient with Diabetic Ketoacidosis: A Unique Case and Review of Literature

**DOI:** 10.1155/2017/6307361

**Published:** 2017-03-02

**Authors:** Kamolyut Lapumnuaypol, Sanna Fatima, Pradhum Ram, Gemlyn George, Antoinette Climaco

**Affiliations:** ^1^Department of Internal Medicine, Einstein Medical Center, Philadelphia, PA, USA; ^2^Department of Infectious Disease, Einstein Medical Center, Philadelphia, PA, USA

## Abstract

*Haemophilus influenzae* is a Gram-negative bacillus commonly known to cause upper respiratory tract infections. Skin and soft tissue infections are very uncommon. Of these, the majority were associated with necrotizing fasciitis requiring emergent debridement. We report a case of pyomyositis caused by* Haemophilus influenzae* in an adult with diabetes.

## 1. Introduction

*Haemophilus influenzae* is a gram-negative bacillus commonly known to cause upper respiratory tract infections. In the years past, Type b was commonly thought to cause invasive infections, however with increasing vaccinations against the strain, other serotypes particularly type F, have been associated with more recent infections in recent times. Regardless of the strain, skin and soft tissue infections caused by this organism are uncommon and only a handful of cases have ever been reported including both pediatric and adult population [1]. Of these, the majority were associated with necrotizing fasciitis requiring emergent debridement. We present here, a case of pyomyositis caused by nontypeable* Haemophilus influenzae* in an adult female with diabetes.

## 2. Case Presentation

A 67-year-old female presented to the emergency department with sudden onset of nontraumatic left thigh pain and inability to bear weight for 1 hour. She had a medical history of uncontrolled diabetes mellitus, hypertension, dyslipidemia, and hepatitis C infection. She denied any injuries or sick contacts at home. On admission, she was noted to be afebrile. On physical examination, the left thigh was noted to be 6 cm larger than the right, exquisitely tender with no obvious erythema or warmth. Her movement at the hip joint was limited secondary to pain. Physical examination of the knee joint, calf, and ankles was unremarkable. Neurological exam was normal and distal pulses were palpable.

Routine chemistry and hematology revealed white blood cell count of 12,100/*μ*L (polymorphonuclear cells 78% with bands 5%), platelet count of 54 × 10^9^/*μ*L, C-reactive protein of 197 mg/L (normal 0–5 mg/L), lactate of 30 mg/dL (normal <19 mg/dL), and creatine kinase of 59 IU/L (normal 20–168 IU/L). She also had blood glucose of 558 mg/dL with anion gap metabolic acidosis (venous pH of 7.33 anion gap 25) and ketones in the urine. Her clinical presentation was suspicious for a soft tissue infection that likely triggered her DKA. The patient was empirically started on vancomycin, cefepime, and metronidazole. She also received intravenous fluids and intravenous regular insulin infusion for treatment of DKA.

**Figure 1:**
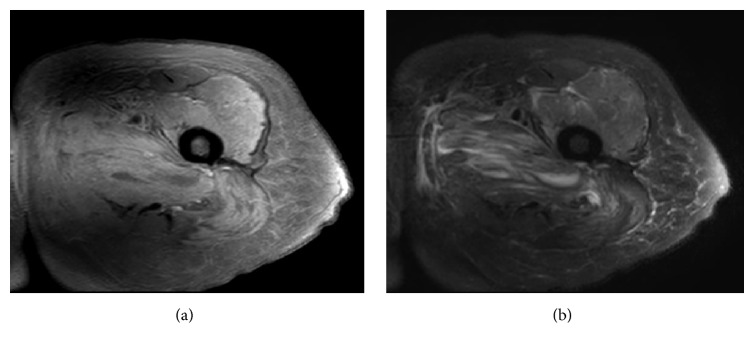
(a) MRI axial T1 with gadolinium, (b) axial T2 without gadolinium showed collection sized 2.9 × 3 cm with intramuscular edema.

An MRI of left lower extremity was obtained that showed intense edema with fluid tracking along the adductor magnus and quadratus femoris muscle and multiple small rim-enhancing fluid collections within the left adductor magnus, which correlated with the site of pain (Figure 1). The largest area of pus collection was 2.9 × 0.8 cm. Surgery and interventional radiology teams were consulted in an attempt to drain the collections. Ultrasound of left thigh was performed, but the pus collection was deemed too small to be drained. The blood cultures grew* Haemophilus influenzae*. The antibiotics were deescalated to ampicillin-sulbactam after the culture results.

The patient was managed with intravenous antibiotics. After 10 days of ampicillin-sulbactam, the patient's pain, swelling, and range of motion improved. C-reactive protein declined gradually from 197 mg/L to 55 mg/L. Her blood glucose was controlled with long-acting insulin and her platelet count normalized prior to discharge. She was discharged to a skilled nursing facility for physical rehabilitation and was to continue amoxicillin-clavulanate for another 2 weeks. Her follow-up visit with primary care physician at 3 months showed that she could ambulate independently.

## 3. Discussion

Pyomyositis is a suppurative infection of skeletal muscle characterized by collection of pus in individual muscle groups. It usually arises from hematogenous spread of an infective focus. It has traditionally been associated with the tropical climate; nonetheless, it has been recognized in temperate climates as well. Tropical pyomyositis occurs in both children and adults, while the temperate pyomyositis occurs primarily in adults. In the past decade, there is an increasing incidence of pyomyositis in a temperate region among children [2]. Most patients with tropical pyomyositis are otherwise healthy without underlying comorbidities; however, majority of the patients in temperate regions are immunocompromised or have other serious underlying conditions [3].

The risk factors associated with development of pyomyositis include immunodeficiency states comprising HIV, diabetes, malnutrition, organ transplantation, and malignancy [3]. Injection drug use and trauma during exercise are some of the other predisposing factors for development of pyomyositis [4, 5]. Given that our patient had uncontrolled diabetes, risk of pyomyositis was increased but it is difficult to ascertain if the pyomyositis was responsible for the development of the ketoacidotic state.

More than 90% of the cases of pyomyositis are caused by* S. aureus* [3]. In a retrospective study of 205 cases in 112 patients with pyomyositis conducted by Chiedozi in Nigeria the most common organism isolated was* S. aureus* followed by* S. pyogenes* [6]. There are very few cases of pyomyositis caused by Gram-negative rods [4, 7–9]. The rare organisms known to cause pyomyositis include* Klebsiella pneumoniae*,* Proteus*, and meninogococcus. To our knowledge, only two cases of* H. influenza* causing pyomyositis in adult and one infant have been described in literature [10, 11].

The patients with pyomyositis most often present with fever and pain at the site of muscle involvement [3]. According to the severity, pyomyositis can be divided into three stages [3]. Stage 1 (between days 1 and 10) is the invasive stage where muscle cramps and low-grade fever are observed, but other physical findings are typically absent. A “woody texture” of muscle may be noted in this stage. The majority of patients present in stage 2 (10–21 days after initial onset of symptoms). This is also called the suppurative stage with abscess formation. The late stage or stage 3 is potentially fatal and is associated with complications such as rhabdomyolysis, endocarditis, septic shock, brain abscess, and other features of disseminated infection. Treatment depends on the stage of disease [3]. Our patient's presentation was compatible with stage 2.

Diabetes is a known risk factor for pyomyositis [12]. In our patient diabetic ketoacidosis and severe pain at the site of muscle involvement led to a diagnosis of pyomyositis. The source of our patient's organism (nontypeable* H. influenzae*) remains unclear, especially in the setting of no recent travel, sick contacts including children, or recent upper respiratory tract infection. Nontypeable* H. influenzae* vaccine in adults has been studied and further clinical trial is warranted to explore clinical benefits [13–15].

## 4. Conclusion

Pyomyositis can be potentially fatal if not diagnosed early enough and treated adequately. Here, we managed a unique and rare case of pyomyositis caused by nontypeable* Haemophilus influenzae.*
